# Interventions to Improve Compliance to Surgical Safety Checklist Use: Before-and-After Study at a Tertiary Public Hospital in Croatia

**DOI:** 10.3390/healthcare13161959

**Published:** 2025-08-10

**Authors:** Jure Krstulović, Zrinka Hrgović, Ante Krešo, Ante Tavra, Ljubo Znaor, Ana Marušić

**Affiliations:** 1 School of Medicine, University of Split, Šoltanska 2A, 21000 Split, Croatia; zrinka.hrgovic@mefst.hr (Z.H.); ante.kreso@mefst.hr (A.K.); ante.tavra@mefst.hr (A.T.); 2 Department of Surgery, University Hospital of Split, Spinčićeva 1, 21000 Split, Croatia; 3 Department of Health Care Quality, University Hospital of Split, Spinčićeva 1, 21000 Split, Croatia; 4 Department of Family Medicine, Split-Dalmatia Health Center, Kavanjinova 2, 21000 Split, Croatia; 5 Department of Ophthalmology, University Hospital of Split, Spinčićeva 1, 21000 Split, Croatia; ljubo.znaor@mefst.hr; 6 Department of Pediatric Disease, Division of Haematology, Oncology, Clinical Immunology and Genetics, University Hospital of Split, Spinčićeva 1, 21000 Split, Croatia; 7 Department of Ophthalmology, University of Split School of Medicine, Šoltanska 2A, 21000 Split, Croatia; 8 Department of Research, University Hospital of Split, Spinčićeva 1, 21000 Split, Croatia; ana.marusic@mefst.hr; 9 Department of Research in Biomedicine and Health, Centre for Evidence-based Medicine, University of Split School of Medicine, Šoltanska 2A, 21000 Split, Croatia

**Keywords:** patient safety, checklist, guideline adherence, quality improvement, safety management, operating rooms, surgery, Croatia

## Abstract

**Background/Objectives:** The World Health Organization’s Surgical Safety Checklist (WHO SSC) is known to reduce surgical complications and mortality, yet its implementation remains inconsistent across institutions. This study evaluated compliance with a modified SSC and assessed the impact of structured interventions to improve adherence at the University Hospital of Split, Croatia. **Methods:** A before-and-after study analyzed a sample of 1437 completed SSCs over four time points between April 2024 and May 2025: the baseline and after three successive interventions (an official letter from the hospital director emphasizing mandatory SSC use, individual meetings with department heads and head nurses reinforcing its importance, and a quality audit review of SSC completeness with leadership). Checklist completeness was assessed across five SSC sections: General Information, Sign In, Time Out, Sign Out, and Staff Info. **Results:** Overall checklist completeness increased from 78.3 ± 8.5% at baseline to 86.3 ± 2.5%, 92.0 ± 3.8%, and 94.7 ± 4.8% after the first, second, and third interventions, respectively (*p* < 0.001). All checklist sections improved significantly: General Info rose from 91.1 ± 6.0% to 98.6 ± 2.6% (*p* < 0.001); Sign In from 85.0 ± 11.4% to 97.0 ± 3.5% (*p* = 0.002); Time Out from 79.0 ± 14.6% to 96.4 ± 6.5% (*p* < 0.001); Sign Out from 70.2 ± 11.2% to 87.7 ± 11.0% (*p* = 0.003); and Staff Info from 70.7 ± 12.9% to 100.0 ± 0.0% (*p* < 0.001). Post hoc testing confirmed significant gains versus baseline for all three interventions (Dunnett’s test), with a further significant improvement between the first and third interventions (Tukey’s HSD, *p* = 0.011). **Conclusions:** Structured, low-cost interventions emphasizing administrative support, education, and communication significantly improved SSC adherence across a large tertiary hospital. This initiative represents a pioneering effort in Croatia and offers a scalable model for similar public healthcare systems aiming to enhance patient safety.

## 1. Introduction

Patient safety is an essential element of high-quality care and a fundamental principle of medical error prevention [1]. In surgical environments, it is especially important to ensure patient safety since harmful incidents, such as retained sponges, mismatched organ transplants, wrong-site surgeries, and unnoticed allergies, can have devastating repercussions on healthcare professionals, patients, and institutions [2]. A significant number of surgical adverse events result from non-technical aspects, including leadership; situation awareness; decision-making; and, most importantly, surgical team collaboration and communication [3,4]. To address these challenges, in 2008, the World Health Organization (WHO) introduced a guideline to improve the safety of surgical patients worldwide [5]. Supported by the WHO, a 19-item Surgical Safety Checklist (WHO SSC) was introduced to improve team communication and care consistency in the perioperative period. A prospective study at eight pilot hospitals in Toronto, New Delhi, Amman, Auckland, Manila, Ifakara, London, and Seattle, comparing pre-intervention and post-intervention periods, demonstrated that, after introduction of the WHO SSC, the rate of inpatient surgical complications dropped from 11.0% to 7.0%, while in-hospital mortality declined from 1.5% to 0.8%, representing an approximately 36% reduction in combined risk of complications and death [6].

The SSC has three checkpoints: (1) prior to the induction of anesthesia (Sign In); (2) prior to the surgical incision (Time Out); and (3) prior to the patient’s departure from the operating theater (Sign Out) [7]. Although the WHO SSC has the potential to significantly reduce preventable mistakes in the theater, mortality, and postoperative complications, the overall compliance with its implementation remains low [8,9,10]. Effective implementation of the SSC requires staff acceptance and adaptation to the hospital’s special requirements and circumstances [11], and the success of the checklist is largely dependent on how it is used [12]. The challenges to the implementation of the WHO SSC are frequently related to institutional environments where the checklist was implemented without sufficient support mechanisms [13]. They include top-down implementation, insufficient team training, and adoption mandated by regulatory bodies and have been linked to failure and little improvement in patient outcomes [14,15]. Also, providers often use the WHO SSC incorrectly or just fill it in postoperatively rather than before the surgery [10,16]. The WHO SSC implementation process is often not standardized, and teams typically do not receive formal training, even if a manual and other resources have been provided [17,18]. Furthermore, the implementation of the SSC faces significant non-technical barriers, primarily rooted in human factors such as poor communication between surgeons and other surgical team members [19,20]; suboptimal team dynamics, often worsened by excessive workload and time pressure [21]; and a lack of checklist ownership and resistance to change [22]. Therefore, a structured and well-planned approach to implementation may serve as an effective model for improving team behavior and safety culture, potentially reducing adverse patient outcomes [23].

The aim of this study was to assess WHO SSC compliance after a series of education and training interventions for effective implementation of the WHO SSC at the University Hospital of Split, which is a part of a public healthcare system with full insurance coverage for health users.

## 2. Materials and Methods

### 2.1. Setting

The University Hospital of Split is the second largest tertiary-level hospital in Croatia and the leading health institution in southern Croatia. It provides specialized and highly complex medical services to over 1 million people in the Dalmatian region and the nearby islands, as well as to more than 3.5 million tourists during the summer months, primarily in Split-Dalmatia County. As an academic teaching hospital affiliated with the University of Split School of Medicine, it plays a significant role in the education of medical students, residents, and other healthcare professionals [24].

The surgical disciplines of the University Hospital of Split are highly developed and encompass a wide range of specialized departments. The General Surgery Clinic is recognized as a Croatian Reference Center for Enhanced Recovery After Surgery (ERAS) [25]. At the same time, the Pediatric Surgery Clinic holds the status of a Croatian Reference Center for Minimally Invasive Surgery [26]. In addition, the hospital features dedicated departments for various branches of surgery, including neurosurgery, maxillofacial surgery, and otorhinolaryngology, as well as specialized departments for urology, gynecology, and ophthalmology. The Department of General Surgery is the largest surgical department, encompassing a multidisciplinary approach with divisions in abdominal surgery, vascular surgery, thoracic surgery, plastic surgery, trauma, and orthopedic surgery [27]. The above-mentioned surgical departments in the University Hospital of Split perform more than 14,700 elective surgical procedures annually. It features 25 operating theaters, of which some are hybrid operating theaters, and is committed to patient safety, evidence-based practice, and surgical innovation. Also, there are a total of 583 hospital beds across the surgical departments. Beyond their work in education and provision of high-quality clinical care, the departments are heavily involved in clinical research and quality improvement initiatives, including the implementation and monitoring of the WHO SSC.

### 2.2. WHO SSC Implementation

The introduction of the WHO SSC at the University Hospital of Split began in late 2022. Up until then, a brief, unmodified version of the checklist had been used only at the Department of General Surgery. The first version of the modified WHO SSC was sent via email to all surgical department heads across relevant hospital units. These department heads were tasked with consulting their teams, comprising surgeons and scrub nurses, to gather feedback and suggestions. Team members were encouraged to submit their comments and proposals via a designated email address at the Department of Health Care Quality. These submissions were then systematically reviewed by the coordinating team. Where necessary, further clarification or revision was undertaken based on the content of the proposals. In addition to this written feedback process, a series of meetings were held to align perspectives, resolve inconsistencies, and reach consensus on key elements of the checklist. These discussions played a crucial role in refining the content and ensuring multidisciplinary agreement prior to implementation. The final version of the SSC was thus developed through a combination of written correspondence, departmental consultations, and structured consensus meetings. After several weeks, a version of the SSC that met all the specified requirements was created (Supplementary Figure S1).

The WHO SSC Implementation Manual [28] allows for adaptations to the specific needs and conditions of different clinical settings, allowing for flexibility in its application across diverse healthcare environments. In our institution, the checklist was modified to include the precise recording of key time points during the surgical process: the time of patient entry into the operating room, initiation of anesthesia, start of surgery, end of surgery, end of anesthesia, and patient exit from the operating room. These additions were important for a systematic analysis of operating room utilization. Furthermore, we expanded the section documenting the patient’s name and date of birth, the number of the operating room, the date and time of surgery, type of anesthesia, type of surgery, and name of the department (“General Info”), as well as a section documenting surgical team members (“Staff Info”), emphasizing the inclusion of signatures from responsible personnel, specifically the lead surgeon and the head of the anesthesia team. These modifications were made to enhance both the accountability and the traceability within the surgical workflow. On 22 March 2023, a pilot test of the modified checklist was conducted in the operating theaters at the Ophthalmology Clinic. The checklist was used during four surgical procedures, and it was concluded that less than four minutes is sufficient to complete the checklist correctly. On 28 March 2023, a meeting about the start of the implementation of the SSC was held at the Directorate of the University Hospital of Split. The invitation for the meeting was sent to all the department heads and head operating room nurses. In the invitation, there was a note that if they were unable to attend, they were asked to send a representative from the department so that the information could be passed on to all surgical departments. All 24 members of the surgery staff participated in the meeting, either in person or through their designated delegates. They were shown a presentation outlining the correct procedure for completing the SSC, with all steps clearly detailed and explained. The presentation also emphasized the critical importance of accurate and thorough checklist completion as a key component of patient safety and quality surgical care. During the meeting, the participants were also shown a YouTube video entitled “How To Do The WHO Surgical Safety Checklist” [29]. A written standard operating procedure (SOP) was also given to all meeting participants (Supplementary File S1), describing precisely how to fill out the SSC and specific responsibilities for different parts of the SSC. According to the SOP, the Checklist Coordinator (CC) is responsible for completing the SSC. The coordinator is typically an operating room technician or a circulating nurse, but may be any team member designated by the surgical team leader. The CC communicates with team members in the prescribed order, guiding the team throughout all questions and circling their responses. The CC is required to circle only one of the predefined answers on the SSC form for each of the questions. Finally, the SSC becomes part of the patient’s paper-based medical record. Our modified SSC is designed in such a way that no question can be left unanswered, because each of the individual sections/questions has an option to be answered with “yes”, “no”, or “not applicable”. To emphasize the importance of accurate medical documentation, the SOP cites national legislation that obliges healthcare professionals to maintain medical records properly. As the SSC is an integral part of the patient’s medical record, the CC is personally accountable for its accurate and complete documentation, in accordance with the Croatian Health Data and Information Act (Official Gazette 14/19). After the meeting, an email with the presentation, SOP, and SSC form was sent to all attendees, setting the implementation date for the checklist as 1 April 2023.

### 2.3. Study Design

In this before-and-after study, three authors (JK, ZH, and AT) collected the data on compliance and completeness of the SSC during the first year after the implementation of the SSC, which was from June 2023 to March 2024. The date of operation, procedure type, elective surgery, type of anesthesia, and adherence to the SSC (General Info, Sign In, Time Out, Sign Out, and Staff Info) were collected for 519 discharged surgical patients. The inclusion criterion for data analysis was the completion of the checklist, defined as one in which all questions had to be answered.

After the first year, we introduced three interventions in 4-month periods. Every four months, we analyzed whether compliance with the SSC had improved. The first intervention, conducted in April 2024, was an official letter from the hospital director that underscored the necessity of completing the SSC as a mandatory patient safety measure that must be followed. The second intervention, conducted in August 2024, involved individual meetings with each department head and the head nurse, during which the information from the director’s letter was repeated, and the importance of properly completing the SSC was once again emphasized as an essential component of patient safety during surgical procedures. The final, third intervention, conducted in December 2024, occurred during the routine annual internal audit of each surgical clinic conducted by the Department of Health Care Quality, which covered all standard evaluation elements. As part of this audit, the completeness of the SSC was reviewed with department heads, with particular emphasis on the importance of proper completion and the need to strive for 100% adherence to the SSC protocol. During each intervention, organization of additional training was consistently offered to the department head or the head nurse if they considered that additional training of the staff on proper SSC implementation was necessary. Regarding the sample size, the target population included 14,700 elective surgical procedures performed at the University Hospital of Split from March 2023 to March 2024. As in similar studies on this topic [30], the sample size calculation, based on a 95% confidence level and a ±5% margin of error, indicated that a minimum of 357 SSCs would be sufficient to achieve statistical representativeness for the target population. Across the four implementation phases, we adopted a methodological approach of randomly reviewing approximately 10% of all surgical cases across the four implementation phases. This sampling percentage was applied to medical records to ensure a representative and proportionally distributed insight across all surgical departments. We reviewed SSCs for 1457 patients, which corresponds to about 10% of the total 14,700 surgical cases performed during the study period. This approach allowed us to obtain both a representative and sufficiently large sample. Thus, we analyzed a total of 1457 SSCs, which is over four times the required sample size. That supports the reliability of our conclusions.

### 2.4. Data Collection

Data collection was carried out by JK, ZH, and AT. An email was sent by the Department of Health Care Quality (JK) to all relevant organizational units requesting the submission of medical documentation to review medical records for the purposes of conducting a retrospective quality assessment of the department’s work. A standardized selection process was applied by administrative personnel within each department, focusing on the medical records of patients who had undergone elective surgical procedures. To minimize potential bias, the hospital departments were not informed in advance regarding the specific elements of documentation that would be subject to evaluation. The selection of records was carried out centrally and independently within the surgical departments by the administrative personnel. The SSC was available exclusively in paper format and constituted an integral component of each patient’s medical record.

The analysis was conducted at four time points (Supplementary Figure S2). The first analysis followed the introduction of the SSC: a total of 519 patient medical histories were analyzed for the periods June–December 2023 and January–March 2024.

Analysis after the first intervention—The intervention involved a letter from the Director of the University Hospital Center Split emphasizing the importance of completing the SSC. A total of 338 patient medical histories were analyzed for the period April–July 2024.

Analysis after the second intervention—This intervention included a notification to the department heads, highlighting the importance of completing the SSC and reminding them of the Director’s letter. A total of 412 patient medical histories were analyzed for the period August–November 2024.

Analysis after the third intervention—In addition to a regular internal audit, this intervention included a discussion with the department heads, emphasizing the importance of completing the SSC and providing information on the current completion. After this third intervention, 168 patient medical histories were analyzed from mid-March to April 2025.

All documentation was retrieved manually from printed surgical records that were delivered to the Department of Health Care Quality. From each delivered medical record, the SSC form was extracted and evaluated for completeness. Data were entered into a spreadsheet and verified independently by two reviewers (ZH and AT). Any discrepancies were resolved by consensus with a third reviewer (JK). This approach ensured high data reliability and allowed for a structured assessment of compliance trends following each institutional intervention.

### 2.5. Statistical Analysis

Descriptive statistics were used to summarize patient demographics and SSC completion across different surgical departments and intervention periods. SSC completeness was evaluated for each of the five predefined sections (General Information, Sign In, Time Out, Sign Out, and Staff Info). Each section consisted of a different number of checklist items, which all had to be answered. The completeness was expressed as the percentage of items correctly filled out per SSC section. For each time point, the average completeness (mean percentage) was calculated across all checklists. Percentage-based completeness was chosen instead of raw scores to account for differences in the number of items per section, allowing for standardized comparison across checklist sections and time points.

To assess the effect of each intervention on checklist compliance, a one-way analysis of variance (ANOVA) was performed using the intervention group (baseline, first, second, and third) as the independent variable and the checklist completeness scores as dependent variables. The rationale for using the one-way ANOVA was that data were not repeated measurements from individual patients, so we did not have subject-paired data, as checklist records were derived from different cohorts. Given the large sample size, the assumption of normality was considered sufficiently met based on the Central Limit Theorem. Type III Sum of Squares was applied due to unequal group sizes, and post hoc pairwise contrasts were conducted using Dunnett’s test versus baseline and Tukey’s HSD for all treatment-to-treatment comparisons. A *p*-value < 0.05 was considered statistically significant. All analyses were performed using JASP Team (2024) (Version 0.19.3).

### 2.6. Ethical Considerations

Approval for this study was obtained from the Ethics Committee of the University Hospital of Split (protocol code 2181-147/01-06/LJ.Z.-24-02, date of approval: 29 April 2024). The study was conducted according to the principles outlined in the Declaration of Helsinki.

## 3. Results

A total of 1437 clinical checklist records were retrieved from the control list archive and included in the analysis. The median age of patients was 53.9 years (interquartile range, 22–84 years). Of the total patient population, 54.3% were male and 45.7% female.

Documentation of the operating room number was present in 84.8% of the checklists, and the procedure date was recorded in 97.9% of cases. Among the checklists that contained information on anesthesia type, 21.1% of procedures were performed under local anesthesia and 78.9% under general anesthesia. About a third (33.4%) of the checklists lacked documentation regarding the type of anesthesia used.

According to the type of surgical procedure (Table 1), general surgery procedures were the most frequently performed, accounting for 19.5% of all cases, followed by trauma and orthopedics (14.5%) and pediatric surgery (12.1%). Ophthalmology and oral and maxillofacial surgery were rarest, with 7.8% cases for each.

Overall, checklist completeness improved over time, with the total score and the score of all five SSC sections significantly higher at the end of Intervention 3, in comparison to the baseline (Table 2). While the scores for individual SSC sections increased gradually over time, the Staff Info section reached almost total completeness after the first intervention. Post hoc analysis of the total scores at different intervention points showed that there was no significant difference between the first and second interventions (*p* = 0.166) or between the second and the third interventions (*p* = 0.631). However, the first and the third interventions differed significantly from each other (*p* = 0.011), indicating that the third phase produced a further improvement beyond that seen after the first phase (Supplementary Table S1).

**Figure 1:**
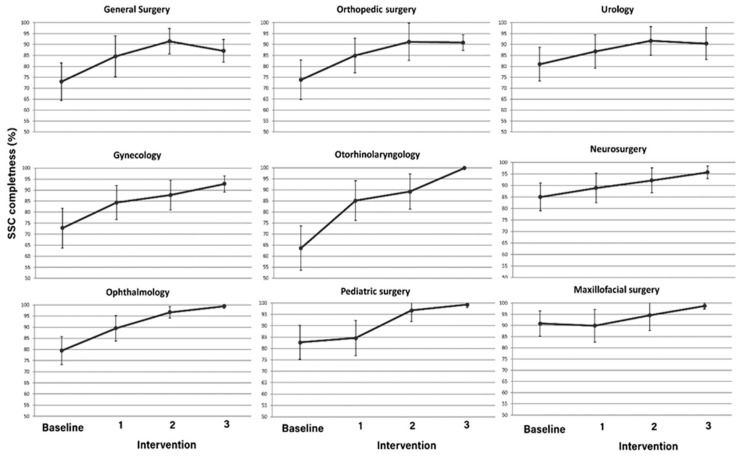
Average checklist completion (% ± standard deviation) by surgical department at baseline and following each intervention phase.

A consistent pattern of improvement in checklist completeness was observed across all surgical departments following the interventions (Figure 1). While certain specialties, such as maxillofacial surgery and neurosurgery, already exhibited high levels of checklist adherence at baseline, others showed marked progress over time. Notably, otorhinolaryngology demonstrated the most substantial improvement, with checklist completeness rising steadily throughout each intervention phase. This department, which started with one of the lowest baseline adherence rates, achieved near-complete compliance by the final phase, reflecting the effectiveness of the implemented measures. Overall, the trend across departments suggests a sustained enhancement in adherence to safety protocols, underscoring the impact of targeted interventions on improving surgical safety culture.

## 4. Discussion

Our before-and-after study aimed to improve compliance with the modified WHO SSC from baseline levels to a target of 100% compliance. While the interventions did not fully achieve the target except in the “Staff Info” part, the findings suggest a positive trend toward increased SSC usage following the interventions. Also, our findings demonstrate a consistent, statistically significant enhancement in checklist completion across every domain throughout the study.

The most significant impact was observed after the third intervention, which included a discussion with the department heads, emphasizing the importance of completing the SSC and providing information on the current completion. Although a direct causal relationship between the interventions and improved compliance cannot be definitively established, the structured involvement of the Department of Health Care Quality, with the full support of the Hospital Directorate, has played a meaningful role in enhancing adherence to the WHO SSC. These results indicate that sustained improvement in checklist compliance requires continuous supervision and the implementation of diverse, systematically planned interventions tailored to the clinical context to achieve the desired result.

The most consistent and statistically robust gains were observed in the “Staff Info” component across nearly all specialties. Following the initial intervention by the hospital director, which emphasized the importance of proper documentation, the Staff Info section showed an immediate improvement in completion compared to other parts of the checklist. As this section is solely the responsibility of the SSC coordinator, who is usually a room circulating nurse, and does not depend on input from other team members, we believe this contributed to its exceptionally high completion from the baseline.

Furthermore, our study found a high completeness in the “Sign In” and “Time Out” sections, with the statistically significant highest levels of compliance observed for items related to patient identity, informed consent, surgical set readiness, equipment availability, and allergies in medical history. These checks are considered among the most critical elements of the checklist, as they address the risk of wrong-patient and wrong-procedure error events, which can cause serious harm to the patient but are entirely preventable [31,32]. Conversely, the Sign Out section was rarely filled out. A potential reason for the low compliance in this field may be inconsistency concerning its targeted timing, which is formally defined as “before the surgeons exit the operating room.” In contrast to other domains, “Sign Out” is not explicitly related to a particular clinical unwanted incident, which may lead to its oversight. Moreover, in our hospital, as we said, the checklist is predominantly filled in by the room circulating nurse, who has several tasks at the conclusion of a surgical procedure, and this may also disrupt the sign-out administration.

Also, following each intervention phase, checklist completion improved significantly across all surgical departments. Despite a smaller sample size, the expected increase in standard deviation did not occur, indicating that the interventions led to more standardized behaviorenhanced training, supervision, and protocol adherence contributed to reduced variability between surgeons and surgical teams.

Our findings are consistent with a recent study conducted at a tertiary-level hospital in India where compliance with the WHO SSC significantly improved after interventions were carried out following a series of rigorous departmental meetings, which served as a structured forum for critical analysis, data-driven decision-making, and collaborative problem-solving with the medical and nursing staff; “Sign In” increased from 45% to 95%, “Sign Out” from 1% to 85%, and “Time Out” from 15% to 90%, demonstrating the effectiveness of awareness programs and training sessions [33]. The evaluation was conducted on 40 surgical procedures, which corresponds to 40 completed Surgical Safety Checklists, whereas in our study, a total of 1437 checklists were evaluated. Also, one study came to the conclusion that a simple and free intervention, such as two-lesson training, significantly improved the proper application of the SSC, with adherence also improving among those who did not participate in the training, probably because of the positive influence of their co-workers’ behaviors [34]. A study conducted in 15 hospitals across Somalia, a country characterized as a resource-limited setting, showed that training interventions, such as practical demonstrations, interactive discussions, and the dissemination of instructional materials, had increased SSC compliance from 37% pre-intervention to 99% post-intervention [35]. A study conducted in Pakistan demonstrated improved compliance with all steps of the SSC following an educational intervention involving a departmental presentation, audit feedback, and implementation guidance—with the most significant improvement (67%) observed in the Sign Out phase [36]. These findings highlight the importance of effective implementation strategies, continuous training, and cultural adaptation to ensure consistent and effective utilization of the WHO SSC. Each healthcare system must identify the most appropriate intervention or combination of interventions tailored to its specific organizational context in order to effectively improve compliance with the Surgical Safety Checklist. For instance, in our previous qualitative study, we identified key barriers to effective SSC implementation, including hierarchical structures that shift responsibility to lower-level staff, administrative burdens, understaffing, inconsistent application, lack of direct training for all team members, and perceptions that certain checklist items are redundant or not applicable across all surgical specialties. Interventions were designed to address these specific challenges for SSC implementation [37]. Our findings correlate with a cross-sectional study conducted in China, where the results indicated that active leadership with experienced operating room nurses, good training for surgical team members, and simplification of the checklist would be the positive factors for effective implementation [38].

It is important to note that in our hospital, a simple/unmodified WHO SSC had already been introduced, but only within the general surgery department. The modified version of the WHO SSC was initially introduced a few years later, and we began assessing compliance nearly a year after that. We believe that the implementation process itself had already laid certain foundations in terms of awareness of the importance of safety that the SSC provides, and that is the reason that our initial percentage of SSC fulfilment was higher than in other studies.

Similar to our study, the “Sign Out” phase, which occurs before the patient leaves the operating room, is consistently reported as the most poorly performed phase of the SSC compared to the “Sign In” and “Time Out” phases [39]. For instance, a study conducted in a tertiary hospital in New Zealand found that the “Sign Out” phase was frequently neglected, thereby raising the possibility of significant omissions in postoperative treatment [40]. Another study conducted in the UK observed that only 8.8% of operations had the “Sign Out” phase attempted, highlighting a lack of compliance due to standard theatrical operational procedures [41]. The reason for the poor compliance of the “Sign Out” phase could be due to the pressure to transfer patients quickly to the recovery area or insufficient time and healthcare workers to complete the checklist items [42,43].

Compliance levels also differ greatly among surgical settings as a result of training deficiencies and attitudes of the surgical staff [22]. Furthermore, studies indicate that the accessibility, clarity, and adaptation of checklists significantly influence their utilization in clinical settings [44,45]. A qualitative study conducted with surgical team members and administrators showed experiences with modifications to the WHO SSC, highlighting themes of local adaptation, responses to adverse events, and the importance of team involvement in enhancing patient safety and checklist effectiveness [46]. Also, a qualitative study from Harris et al. proposes that adding checklists to established protocols, such as ERAS, might help to improve adherence and decrease complications [47]. Building on the findings of our previous qualitative study, which identified hierarchical dynamics within surgical teams and persistent logistical constraints as key barriers to effective SSC implementation, this follow-up intervention focused on addressing those challenges through a series of structured discussions with key stakeholders and responsible personnel. By actively involving surgical team leaders and hospital management in identifying practical solutions and reinforcing accountability, the intervention aimed to strengthen team cohesion and improve checklist compliance through context-specific organizational adjustments [32]. Similar to our study, a quality improvement study conducted in Brazil demonstrated that the active involvement of decision-makers, alongside team engagement and training, played a key role in improving adherence to the WHO SSC, even though full compliance was not achieved [48]. Such findings align with those of Bashford et al. [49], who observed that a coordinated approach involving hospital management significantly impacted teamwork perceptions and safety climates among surgical staff, contributing to better training outcomes and checklist compliance. Moreover, qualitative research in 20 Australian public hospitals showed that hospital leaders implemented the SSC by employing a range of supportive and sanction-based strategies, demonstrating responsive regulation to gradually improve compliance over time [50].

Finally, although there is strong supporting evidence that SSCs are associated with improved patient outcomes, including lower morbidity and mortality rates, effective implementation and high levels of compliance depend on a complex web of tactics, including education, leadership, team behavior, and cultural transformation in surgical environments [51,52,53].

To our knowledge, this is the first quality improvement study conducted in our institutional setting, providing valuable insights into the level of checklist adherence pre- and post-intervention. Also, we are not aware of similar studies from other hospitals in Croatia or neighboring countries. To the best of our knowledge, this is one of the few studies that systematically assessed the implementation of the SSC across multiple surgical departments within a single tertiary care institution. By including diverse surgical specialties—such as maxillofacial surgery, ophthalmology, pediatric surgery, and otorhinolaryngology—our study offers a comprehensive and context-sensitive evaluation of checklist utilization in routine clinical practice. This multiclinic approach allowed us to identify interdepartmental variations and monitor the effects of targeted interventions over time. Also, this study employed a systematic, staged approach to change, comprising three separate organizational challenges over the course of one year, which allowed for the analysis of compliance patterns. In addition, our study evaluated specific components of the checklist in detail, allowing for identification of specific shortcomings, including, among others, “Sign In” and “Time Out,” and ongoing slower growth in compliance during the “Sign Out” process. One of our biggest strengths is the large sample size (*n* = 1437 checklists), which supports the robustness of the results. Moreover, as we said, the structured implementation of the SSC at our institution was strongly supported by the Department of Health Care Quality and the hospital leadership, which facilitated staff engagement through training sessions, written protocols, and regular follow-up. While these measures undoubtedly contributed to improved adherence, we recognize that local institutional culture, including hierarchical dynamics, administrative expectations, and interprofessional communication norms, may have influenced how the checklist was perceived and applied in daily practice.

We acknowledge several limitations of this study. First, the research was conducted at a single tertiary-level hospital, which limits the generalizability of the findings to other institutions or healthcare settings. Second, the study did not evaluate the impact of checklist adherence on clinical outcomes such as postoperative complications, morbidity, or mortality. While a statistically significant improvement in compliance was observed over the one-year study period, it remains uncertain to what extent this improvement could be sustained without ongoing monitoring, education, and reinforcement efforts. Additionally, the implementation process relied heavily on local leadership engagement and individual staff motivation, introducing potential variability in the fidelity of the intervention. These limitations highlight the need for future studies with more rigorous methodological designs because it is not clear to what extent this process can be maintained without continued surveillance and reinforcement, which is another idea for future research.

## 5. Conclusions

In summary, we were effective in improving adherence to our institution’s WHO SSC. We observed significant increases in checklist compliance after implementing the structured interventions, demonstrating that focused educational and organizational strategies can successfully change surgical team behaviors. Such improvements in checklist use are likely to enhance surgical teamwork and patient safety, highlighting the broader importance of promoting WHO SSC adherence in everyday practice. Notably, this initiative is the first of its kind in a post-communist country and represents a pioneering effort within our hospital. The positive outcomes documented here can inspire and inform other hospitals to undertake comparable interventions to boost checklist compliance and overall surgical safety. Improving adherence to safety checklists is not merely a procedural enhancement—it represents a fundamental commitment to patient safety, clinical accountability, and the continuous pursuit of healthcare quality. Our experience demonstrates that systematic implementation, strong institutional support, and staff engagement can lead to measurable improvements in SSC compliance, an essential foundation for advancing surgical care quality and patient safety, even if direct outcome effects remain to be demonstrated. Therefore, we acknowledge that local cultural and environmental factors, such as organizational hierarchy, communication norms, and institutional climate, influenced checklist adherence, and we propose this as a valuable direction for future research to better understand contextual determinants of implementation success. Also, to support long-term compliance with the SSC, several measures are planned; the checklist will be integrated into the hospital’s electronic health record system, allowing for real-time documentation and easier monitoring; regular audits with feedback to department heads will continue regularly, and training sessions, with additional education available upon request, will be conducted.

This approach can serve as a practical and scalable model for healthcare facilities aiming to elevate standards of care and build resilient, high-reliability systems.

## Figures and Tables

**Table 1:** Types of procedures included in the analysis.

Procedure	No. (%) of Patients
General surgery (abdominal, vascular, thoracic surgery)	279 (19.5)
Trauma and orthopedics	208 (14.5)
Pediatric surgery	172 (12.1)
Otorhinolaryngology (ENT)	120 (8.4)
Ophthalmology	111 (7.8)
Gynecology and obstetrics	155 (10.8)
Neurosurgery	126 (8.8)
Urology	148 (10.3)
Oral and maxillofacial surgery	111 (7.8)

**Table 2:** Surgical Safety Checklist completion (mean % ± standard deviation) by section across intervention phases.

Checklist Section	Baseline	Intervention 1	Intervention 2	Intervention 3	*p*-Value ^a^
Total Average	78.0 ± 8.5	86.3 ± 2.5	92.0 ± 3.8	94.7 ± 4.8	<0.001
General Info	91.1 ± 6.0	95.2 ± 3.7	98.0 ± 1.8	98.6 ± 2.6	<0.001
Sign In	85.0 ± 11.4	90.0 ± 5.8	95.3 ± 1.8	97.0 ± 3.5	0.002
Time Out	79.0 ± 14.6	84.3 ± 8.5	92.6 ± 4.4	96.4 ± 6.5	<0.001
Sign Out	70.2 ± 11.2	75.1 ± 4.8	81.6 ± 10.2	87.7 ± 11.0	0.003
Staff Info	70.7 ± 12.9	100.0 ± 0.0	99.7 ± 0.6	100.0 ± 0.0	<0.001

^a^ One-way ANOVA testing for differences in completeness across the four periods.

## Data Availability

The data are available upon reasonable request from the corresponding author.

## Supplementary Materials

The following supporting information can be downloaded at https://www.mdpi.com/article/10.3390/healthcare13161959/s1: Figure S1: Surgical Safety Checklist form—University Hospital of Split. Figure S2: Data collection flow chart. Table S1: Pairwise statistical comparisons of Surgical Safety Checklist completeness. Supplementary File S1: University Hospital of Split–standard operating procedure for completion of the Surgical Safety Checklist.

## Abbreviations

The following abbreviations are used in this manuscript:

WHO SSC	World Health Organization’s Surgical Safety Checklist
SSC	Surgical Safety Checklist
ERAS	Enhanced Recovery After Surgery
CC	Checklist Coordinator
ANOVA	One-Way Analysis of Variance
