# Supplementary material for: Interventions to Improve Compliance to Surgical Safety Checklist Use: Before-and-After Study at a Tertiary Public Hospital in Croatia
Source: Healthcare (Basel). 2025 Aug 10;13(16):1959. doi: 10.3390/healthcare13161959 (PMC12385942; doi:10.3390/healthcare13161959)
Supplement: Supplementary file 1 [file healthcare-13-01959-s001.zip › Supplementary Figure S1 - Surgical Safety Checklist Form - University Hospital of Split eng..pdf]

Supplementary Figure S1 - Surgical Checklist Form - University hospital of Split

KBC Split

OZ SSC; version 3, in use from 31.3.2023.

SURGICAL SAFETY CHECK LIST

Patient name and surname:<br>Sex **M F**	Date of birth/SSN:	Operating room:<br>Type of anesthesia:	Date of Surgery:<br>Tipe of Surgery: elective / emergency

BEFORE INDUCTION OF ANESTHESIA (SIGN IN)

SSC COORDINATOR

Time of entry into the OR
_______h ______ min

Patient identity confirmed? YES NO

Consent forms filled out and signed?
YES NO

Site of procedure marked?
YES NO N/A

ANAESTHESIOLOGIST/ANAESTHESIOLOGICAL TECHNICIAN

Risk of blood loss greater than 500ml?
YES NO

Blood type determined and required blood doses reserved?
YES NO

Additional venous access provided? YES NO

Thromboprophylaxis?
YES NO

Is the anesthesia equipment functioning properly?
YES NO

Required medications prepared?
YES NO

Allergy history?
YES Specify:______________________________
NO Unknown

Anesthesia start time
_______h ______ min

BEFORE THE FIRST INCISION (TIME OUT)

SSC COORDINATOR

All team members introduced by name and function?
YES NO

Patient positioned correctly?
YES NO

Radiological images prepared for review?
YES NO N/A

SCRUB NURSE

Set of instruments sterile and complete?
YES NO

Number of compresses: _______ N/A

Number of swabs: ________ N/A

SURGEON

Confirmed patient name, location and type of surgery?
YES NO

Antibiotic prophylaxis administered?
YES NO N/A

Appropriate implant material provided?
YES NO N/A

Operation start time
_______h ______ min

BEFORE THE PATIENT LEAVES THE OPERATING ROOM (SIGN OUT)

SCRUB NURSE

Correct number of compresses and swabs:

Before and after body cavity closure?
YES NO N/A

Before patient leaves the operating room?
YES NO

Exact number of instruments and needles?
YES NO

Problems with equipment during surgery?
YES NO

Problem recorded and reported?
YES NO

Sample taken for PH and/or ICD analysis
YES NO N/A

Sample properly labeled for PHD and/or ICD analysis
YES NO N/A

Note:

SURGEON

End of surgery time
_______h ______ min

Final name of the surgical procedure?
____
____

Was intraoperative X-ray used?
YES NO N/A

ANAESTHESIOLOGIST

End of anesthesia time
_______h ______ min

Patient is placed in:

Recovery	YES	NO
Intensive care unit	YES	NO

Time of departure from the operating room
_______h ______ min

1st assistant:

2nd assistant:

Resident anesthesiologist:

Scrub nurse:

SSC Coordinator:

Anesthesiologist technician:

ANESTHESIA TEAM LEADER SIGNATURE:

SURGICAL TEAM LEADER SIGNATURE:

SSC entered in: HIS Protocol
Signature:
