# Supplementary material for: Interventions to Improve Compliance to Surgical Safety Checklist Use: Before-and-After Study at a Tertiary Public Hospital in Croatia
Source: Healthcare (Basel). 2025 Aug 10;13(16):1959. doi: 10.3390/healthcare13161959 (PMC12385942; doi:10.3390/healthcare13161959)
Supplement: Supplementary file 1 [file healthcare-13-01959-s001.zip › Supplementary File S1 - University Hospital of Split - SOP SSC_ EN.pdf]

Machine Translated by Google

KBC Split	Name of the instruction: **PROCEDURE FOR FILLING OUT SURGICAL FORM CHECKLISTS**		
Code: RU-SUK-04	Edition: 1	In effect from: 27.3.2023.	Page: 1/8

CONTENT:

1. PURPOSE

2. SCOPE OF APPLICATION

3. RESPONSIBILITIES AND AUTHORITIES

4. DEFINITIONS

5. ACTIONS, MEANS AND METHODS

5.1. Preoperative: before the patient is put under anesthesia (sign in)

5.1.1. Patient identity verification

5.1.2. Verification of signatures of required consents:

5.1.3. Anaesthesia safety check

5.2. Preoperative procedures before surgical incision - TIME OUT 5.3. Postoperative procedures - SIGN OUT 5.4. Data entry into the surgical protocol and BIS 6. LINKS TO OTHER DOCUMENTS

7. FORMS

8. ATTACHMENTS

9. REFERENCE

10. OVERVIEW OF CHANGES

	Ime i prezime	Datum ovjere	Potpis
Izradio:	Jure Krstulović, dr. med.<br>Odjel za osiguranje i unaprjeđenje kvalitete zdravstvene zaštite	23.3. 2023.	[signature]
Pregledali:	doc. dr. sc. Slavica Dajak, dr. med.<br>Pomoćnica ravnatelja za kvalitetu zdravstvene zaštite i nadzor	23. 03. 2023.	[signature]
	Izv. prof. Ljubo Znaor, dr. med.<br>Klinika za očne bolesti	23. 3. 23.	[signature]
	dr. sc. Miro Jukić, dr. med.<br>Klinika za dječju kirurgiju	24. 3. 2023	[signature]
	Ante Mihovilović, dr. med.<br>Zavod za maksilofacijalnu kirurgiju	23.03. 2023.	[signature]
Odobrio:	Prof.dr.sc. Julije Meštrović, dr.med<br>Ravnatelj KBC Split	24.03. 2023.	[signature]

Machine Translated by Google

1. PURPOSE

The Surgical Checklist (hereinafter: KKL) represents a quality standard by which significantly improves the outcome of the surgical procedure, patient safety and reduces complications in patients undergoing surgery. The KKL also serves as an instrument for improving communication and teamwork, which all together further improves safety and efficiency surgical procedure, and thus greater safety for the patient.

This procedure describes the method of managing KKL based on the World Health Organization surgical checklist. healthcare organizations.

2. SCOPE OF APPLICATION

KKL is applied in all healthcare organizational units where surgical procedures are performed. procedures at KBC Split.

3. RESPONSIBILITIES AND AUTHORITIES

To establish an operational security clearance process, as well as to properly complete and Managers are responsible for recording all steps of the operational security review process. surgical and anesthesiological activities and head nurses of departments where surgical procedures are performed procedures.

All members of the surgical and medical teams are authorized and responsible for the application of KKL during the surgical procedure. anesthesiology team (surgical team leader, surgical assistant 1, surgical assistant 2, medical nurse, operating room technician, auxiliary nurse/instrument nurse, head of the anesthesia team, resident in anesthesia, anesthesia technician) in a way that is described further in this instruction. The KKL is first completed in paper form and only then charged person from the Surgical Team Leader enters all data into the BIS. NOTE: The Surgical Checklist is an integral part of patient treatment data, so the person is responsible for managing surgical checklists personally responsible for correctly and completely entering the required data, which is why in the event of Violation of this obligation is equally liable as in the case of improperly maintained medical documentation. and the consequences that may arise from such a failure. The Law on Data and Information in Health NN 14/19.

4. DEFINITIONS

Machine Translated by Google

Surgical team leader: The surgical team leader is a specialist in a particular branch of surgery (surgeon). He is responsible for the work and functioning of the entire surgical team during the duration of the operation. procedure, is superior to all members of the surgical team participating in the operation.

Assistant 1: The first assistant is either a specialist or a resident in a particular branch of surgery who works in accordance with with their competencies as determined by the head of the surgical team.

Assistant 2: The second assistant is usually a resident but can also be a specialist in a specific field. surgery who works in accordance with his/her competencies as directed by the head of the surgical team.

Surgical nurse: is part of the surgical team - works under the instructions of the surgical team leader team. The tasks of the instrument nurse are the proper use and handling of surgical instruments and surgical equipment during the surgical procedure. It is the duty of the operating room nurse to adhere to principles and rules of asepsis, maintains cleanliness and order, and recognizes signs of contamination and cessation sterility. She is also responsible for the completeness of the materials (number of compresses, swabs, surgical instruments and other materials) used during the surgical procedure in the manner described in this manual.

KKL Coordinator: is responsible for fulfilling the KKL. It is important to emphasize that the control coordinator The list is usually **an operating room technician or a medical assistant. nurse/instrument operator,** but if necessary, it can be any member of the team participating in the operation the surgical team leader's instructions. The coordinator communicates with the team members in the order in which indicated on the KKL. Parts of the KKL are completed independently and confirmed before induction of anesthesia the truthfulness of the data with your signature.

Head of the anesthesia team: The head of the anesthesia team is a specialist in anesthesiology and resuscitation. He is responsible for the work and functioning of the entire anesthesiology team at performing anesthesia procedures during the duration of the surgical procedure. He is superior to all members of the anesthesia team participating in the surgical procedure.

Anesthesiology resident: a resident participates in anesthesia procedures in accordance with their competencies as prescribed and under the supervision of the head of the anesthesia team.

Machine Translated by Google

Anesthesia Technician: The tasks of an anesthesia technician are to prepare the space, equipment, and materials for performing anesthesia procedures and providing or participating in the application anesthesia procedures to the patient as ordered by the head of the anesthesia team.

5. ACTIONS, MEANS AND METHODS

The checklist coordinator is responsible for completing the KKL. The checklist coordinator is auxiliary nurse/instrument technician, but if necessary it can be any member of the team who participates in the operation. The coordinator communicates with the team members in the order indicated on the KKL. Communication between members of the operations team must be effective and based on clear questions and short, clear and loud answers that must be documented by the coordinator process.

The operational security review process is carried out in three parts:

- o Preoperative, before the patient is put under anesthesia (sign in)
- o Preoperative, before the first surgical incision (time out)
- o Postoperative, before the patient leaves the operating room (sign out)

5.1. Preoperative procedures before placing the patient under anesthesia – SIGN IN

In this phase, the coordinator enters the time the patient enters the operating room.

Preoperative procedures include:

5.1.1. Patient identity verification

Upon the patient's arrival in the operating room, the coordinator enters the patient's identification information into the KKL. patient information (name, surname, date of birth and MBO), name of the hall and date. Coordinator verifies the patient's identity by checking the identification bracelet, data on the History illness and verbal confirmation of identity (if possible).

5.1.2. Verification of signatures of required consents:

- o Consent for surgery (separate consent forms are prepared for each surgery) consent)
- o Consent for anesthesia (unique consent)
- o Consent to receive a transfusion (unique consent)

Machine Translated by Google

The coordinator documents the aforementioned check on the KKL.

Remark:

When confirming identity, location of intervention and giving consent is impossible (child or patient who has been deprived of legal capacity) a family member may, before the procedure begins provide information on behalf of the patient. In the event that a family member is not present or in the event of an emergency This part of the operating procedures must be subsequently checked and recorded in writing.

5.1.3. Anaesthesia safety check

Anesthesiologist with anesthesiology resident and/or anesthesia technician, before induction of anesthesia determines and documents: the proper functioning of the anesthesia equipment and adequate preparation of medications

Checks for allergies and the risk of blood loss.

All observations in this area as well as preventive measures taken (venous route, blood doses in preparation, thromboprophylaxis) must be recorded on the KKL.

After the anesthesia safety check is completed, anesthesia induction procedures are performed. and the time of the start of anesthesia is entered on the KKL.

5.2. Preoperative procedures before surgical incision - TIME OUT

This period includes the time interval from induction of anesthesia to surgical incision.

In this phase, the members of the surgical team introduce themselves to the patient: by name, surname, and function.

The patient, if not sedated, can also participate in these checks.

The coordinator is required to confirm whether the patient's position is appropriate for a specific surgical procedure, whether whether (if necessary) basic diagnostic images important for planning and implementation have been set up of a single surgical procedure.

Before skin incision, **nurses/instrument operators** verbally confirm sterility, correctness and completeness of the tools and equipment, and indicate the number of compresses and swabs. Once again before the surgical incision **the surgical team leader** confirms that the correct operation is being performed on the correct patient, on on the correct side of the body, whether the patient received antibiotic prophylaxis 60 minutes before the start of the surgery (if necessary), and whether appropriate installation material has been provided.

Machine Translated by Google

No skin incision should be made until all participants have agreed on all key information. When The incision begins, the time of the start of the operation is entered in the KKL.

5.3. Post-operative procedures – SIGN OUT

This part of the process includes the time interval from the closure of the surgical wound to the patient's discharge from the hospital. operating room, must be performed before the patient leaves the operating room.

Nurses/instrument operators determine the complete number of compresses, swabs, surgical instruments and other materials used during the operation. They summarize whether there were any problems with equipment and instruments. This information is documented and reported to the responsible person in in case of any problems. The KKL documents whether the sample was taken and correctly labeled for analysis (tissue preparation or microbiological sample).

The surgical team leader, at the end, confirms that the operation has been performed, states the final name and time of completion of the surgical procedure.

Depending on the course of the operation and the patient's condition, **the head of the anesthesia team** gives instructions for Postoperative care is recorded in the KKL and the time of completion of anesthesia is entered.

The heads of the surgical and medical departments confirm with their signatures that the KKL has been completed correctly and truthfully. **the anesthesiology team.**

After the patient leaves the operating room, **the coordinator** enters the time of departure into the KKL. operating rooms.

5.4. Data entry into the operational protocol and BIS

Upon completion of the surgical procedure, the surgical protocol is written and the KKL is filled out. hospital information system (BIS), a person designated by the manager is responsible for entering data surgical team. The surgical protocol must contain the following information: patient's name and surname, patient identification number, first and last name of the persons who participated in the surgical team, type anesthesia techniques, name and surname of the anesthesia specialist, surgery performed, type surgery (DTS procedure and descriptive), preoperative diagnosis (ICD.10 and descriptive), postoperative diagnosis, intraoperative pathology specialist in cases where intraoperative biopsy examination, complications if any, tissues removed, intraoperative description of procedures (such as opening and closing a surgical wound, obtaining a transplant, dissecting tissue,

Machine Translated by Google

tissue removal, device implantation) performed by others instead of the main surgeon (e.g. residents),

During the printing of the operation protocol, it is mandatory to enter in the designated columns: time patient's arrival in the operating room, time of surgery start, time of surgery end, time the patient leaves the operating room, time the anesthesia starts and time it ends anesthesia. **The data entered into the BIS must be identical in paper and electronic form. shape.**

Upon completion of filling in the data, the paper version of the KKL is stored in the medical history, signed from the coordinator who filled it out.

6. LINKS TO OTHER DOCUMENTS

RU-SUK-03 Identification of hospitalized patients

7. FORMS/RECORDS

Completed Surgical Checklist

Operation protocol

8. ATTACHMENTS

OZ - KKL

9. REFERENCE

Implementation Manual WHO Surgical Safety Checklist 2009

10. OVERVIEW OF CHANGES

Edition 1
