# Supplementary material for: Interventions to Improve Compliance to Surgical Safety Checklist Use: Before-and-After Study at a Tertiary Public Hospital in Croatia
Source: Healthcare (Basel). 2025 Aug 10;13(16):1959. doi: 10.3390/healthcare13161959 (PMC12385942; doi:10.3390/healthcare13161959)
Supplement: Supplementary file 1 [file healthcare-13-01959-s001.zip › Supplementary Table S1 - Pairwise statistical comparisons of surgical safety checklist completeness.pdf]

Supplementary Table S1. Pairwise statistical comparisons of surgical safety checklist completeness: Dunnett tests versus baseline and Tukey HSD among interventions

Comparison	Method	Mean Diff (%)	Test Stat (t)	p-value	95 % CI	Significant
Intervention 1 vs Baseline	Dunnett	8.30	3.326	—	—	Yes
Intervention 2 vs Baseline	Dunnett	13.61	5.454	—	—	Yes
Intervention 3 vs Baseline	Dunnett	16.60	6.656	—	—	Yes
Intervention 1 vs Intervention 2	Tukey HSD	5.31	—	0.1659	–1.45 to 12.07	No
Intervention 1 vs Intervention 3	Tukey HSD	8.31	—	0.0112	1.55 to 15.06	Yes
Intervention 2 vs Intervention 3	Tukey HSD	3.00	—	0.6307	–3.76 to 9.75	No

CI = confidence interval;

Dunnett values use a critical $t = 2.709$ ($\alpha = 0.05$, $k = 4$, $df = 196$); Tukey HSD provides adjusted p-values for all pairwise contrasts.
"—" indicates not applicable.
