# Supplementary figures and images for: Interventions to Improve Compliance to Surgical Safety Checklist Use: Before-and-After Study at a Tertiary Public Hospital in Croatia
Source: Healthcare (Basel). 2025 Aug 10;13(16):1959. doi: 10.3390/healthcare13161959 (PMC12385942; doi:10.3390/healthcare13161959)

**Supplementary Figure S2. Data Collection Flow Chart**

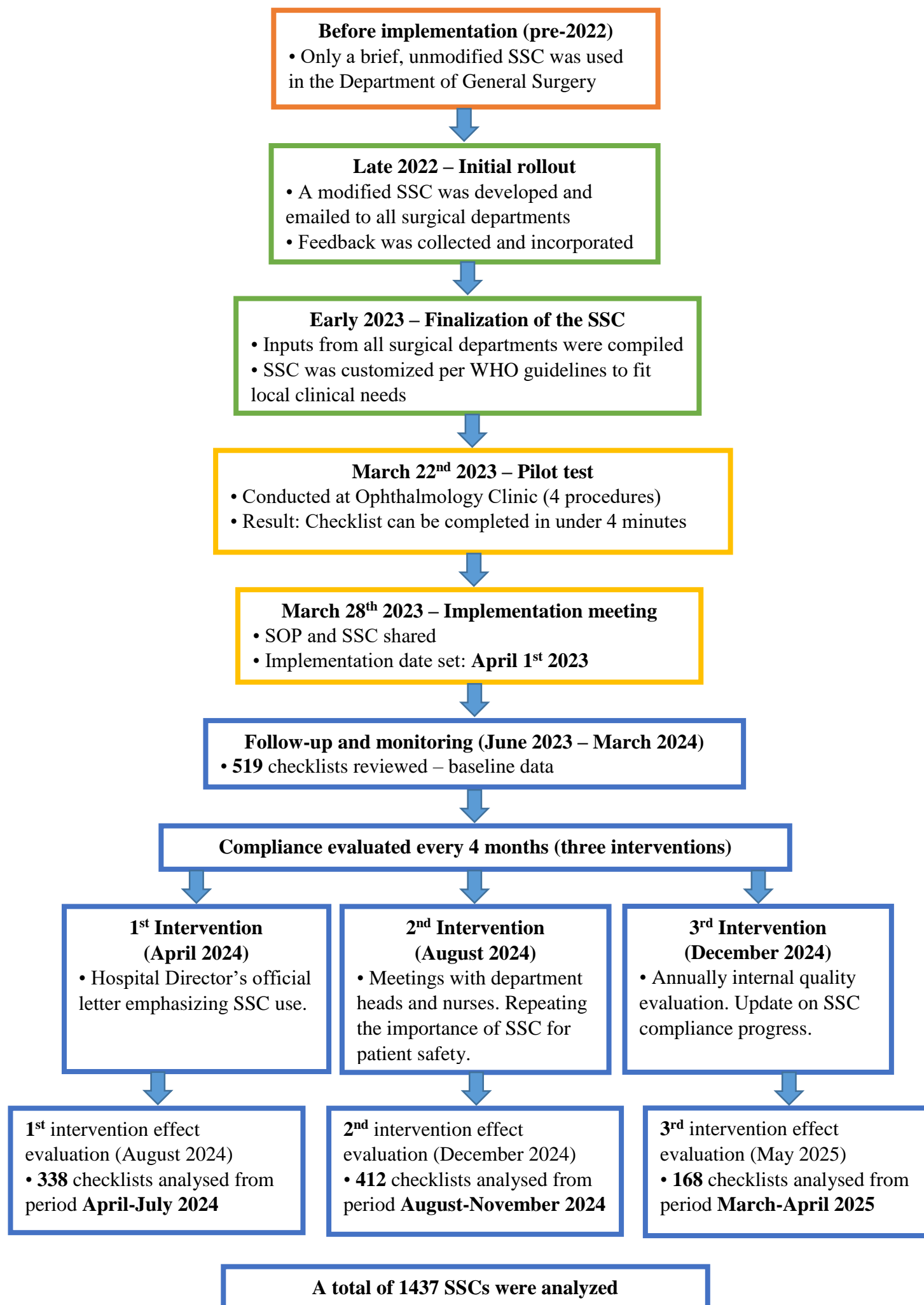

Supplement: Supplementary file 1 [file healthcare-13-01959-s001.zip › Supplementary Figure S2. Data Collection Flow Chart-1.pdf]
